# Metachronous Pancreatic Metastasis of Myxoid Liposarcoma Successfully Treated With Robotic Spleen‐Preserving Distal Pancreatectomy With Splenic Vessels Resections: A Case Report

**DOI:** 10.1111/ases.70069

**Published:** 2025-04-22

**Authors:** Yumi Sota, Kosei Takagi, Motohiko Yamada, Tomokazu Fuji, Kazuya Yasui, Takeyoshi Nishiyama, Yasuo Nagai, Noriyuki Kanehira, Akari Masunaga, Toshiyoshi Fujiwara

**Affiliations:** ^1^ Department of Gastroenterological Surgery Okayama University Graduate School of Medicine, Dentistry, and Pharmaceutical Sciences Okayama Japan

**Keywords:** myxoid liposarcoma, pancreatic metastasis, robotic surgery

## Abstract

Pancreatic metastasis of myxoid liposarcoma (MLS) after primary resection is extremely rare. Herein, we present a case of metachronous pancreatic metastasis of MLS that was successfully treated with robotic spleen‐preserving distal pancreatectomy (SPDP) using the Warshaw technique. A 60‐year‐old woman underwent radical resection of a 25‐cm MLS in the right thigh after receiving neoadjuvant radiotherapy. The patient developed a 6‐cm solitary pancreatic metastasis of the MLS 2 years later. Because no other distant metastases were detected, robotic SPDP (Warshaw technique) was performed. The operative time was 140 min with minimal blood loss. Follow‐up at 3 months showed no recurrence. To our knowledge, this is the first report of a case of metachronous pancreatic metastasis of MLS successfully treated with robotic SPDP. Curative resection using minimally invasive surgery should be performed for solitary pancreatic metastases from MLS.

## 1 Introduction

Liposarcoma is one of the most common subtypes of soft tissue sarcoma (STS), accounting for approximately 15%–20% of all STS cases. It is classified into five histological subtypes: well‐differentiated, dedifferentiated, myxoid (MLS), pleomorphic, and myxoid pleomorphic liposarcomas [1]. MLS is the most common histologic subtype, accounting for approximately 30% [2, 3]. Although MLS is usually treated with radical resection combined with radiotherapy, approximately 30% of the patients develop local recurrence or distant metastases, including multiple lung and soft tissue metastases [4]. Pancreatic metastasis of MLS is extremely rare, with only two cases reported in the English literature [5, 6]. Moreover, no studies have reported using minimally invasive surgery for pancreatic metastasis of MLS.

Here, we present a rare case of metachronous pancreatic metastasis of MLS that was successfully treated with robotic spleen‐preserving distal pancreatectomy (SPDP) using the Warshaw technique.

## 2 Case Presentation

**FIGURE 1:**
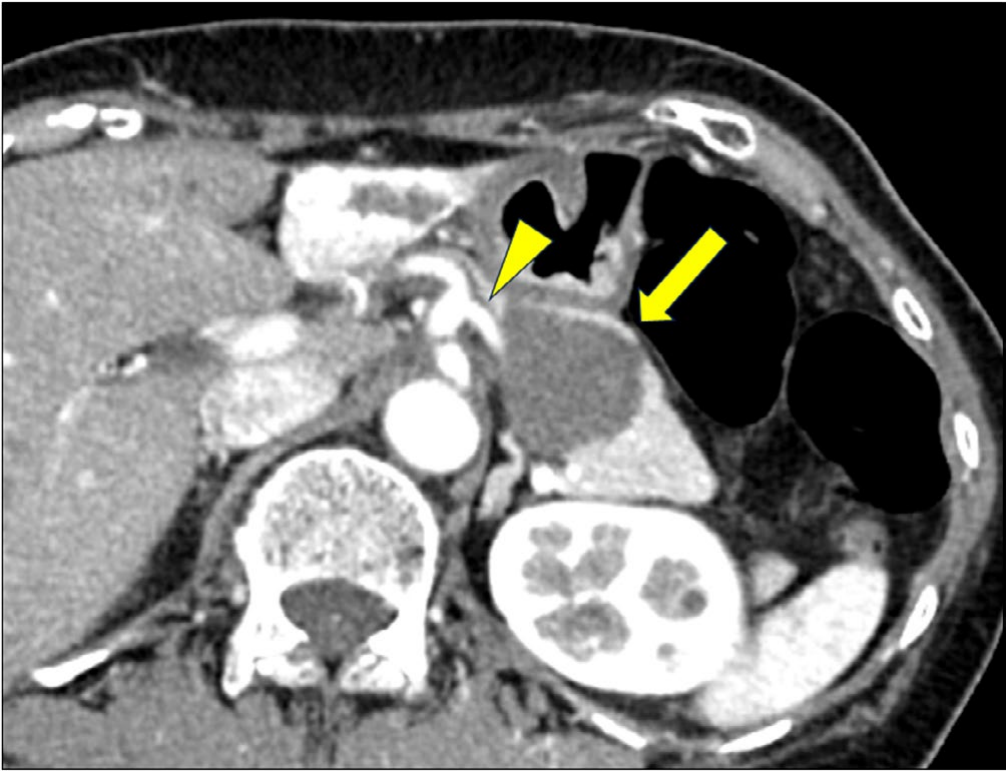
Contrast‐enhanced computed tomography shows a cystic tumor with gradual contrast enhancement in the body of the pancreas (arrow). The tumor was in contact with the splenic artery (triangle).

A 60‐year‐old woman was diagnosed with a primary MLS in the right thigh, measuring 25 cm in diameter (Figure S1). The patient had no significant medical history, including any other malignant diseases. After receiving neoadjuvant radiotherapy (50 Gy), the patient underwent radical resection of the primary lesion. Postoperatively, the patient received no adjuvant therapy and was followed up with radiological examinations every 4 months. Magnetic resonance imaging revealed a 25‐mm tumor in the pancreatic body 2 years after the surgery (Figure S2). Three months later, contrast‐enhanced computed tomography revealed an enlarged 60‐mm tumor in the pancreatic body with gradual contrast enhancement (Figure 1). Magnetic resonance imaging revealed hypointense lesions on T1‐ and hyperintense lesions on T2‐weighted sequences of the pancreatic body (Figure S3). Endoscopic ultrasonography revealed a hypoechoic tumor. Moreover, contrast‐enhanced endoscopic ultrasonography demonstrated an isovascular pattern, suggesting an atypical pancreatic tumor such as a metastatic tumor. Fine‐needle aspiration cytology was not conducted, considering the potential risk of tumor seeding. Accordingly, the preoperative diagnosis was solitary pancreatic metastasis of MLS. The differential diagnoses included pancreatic cystic tumors such as mucinous cystic neoplasms and intraductal papillary mucinous neoplasms. Because no other distant metastases were detected, surgical intervention was performed.

Regarding the surgical strategy, there were no signs of tumor invasion into the surrounding tissues or splenic vessels. However, the pancreatic tumor was in contact with the splenic vessels. Robotic SPDP with splenic vessel resection (Warshaw technique) was performed to achieve R0 resection with negative margins.

**FIGURE 2:**
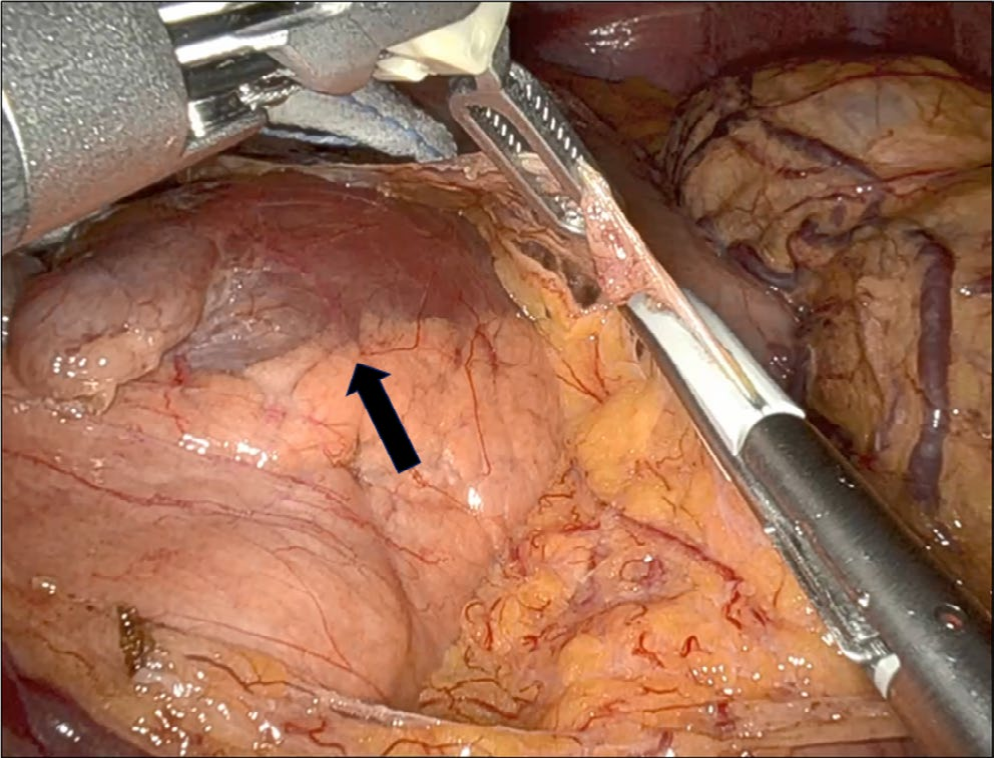
A large tumor in the body of the pancreas (arrow).

This procedure was consistent with our surgical protocol [7, 8]. A da Vinci Xi robotic system (Intuitive Surgical, Sunnyvale, CA, USA) was used. Following division of the gastrocolic ligament, a large tumor was confirmed in the pancreatic body (Figure 2). The pancreatic body was dissected and encircled by the superior mesenteric artery (SMA). The splenic artery was isolated and divided using the posterior approach. A progressive stepwise compression technique with a stapler was used to transect the pancreas into the SMA. Subsequently, the pancreatic body and tail were dissected toward the splenic hilum using a medial approach. A retroperitoneal dissection line was placed in front of the anterior Gerota fascia (level 1) [9]. Finally, the splenic hilum was encircled and transected using a stapler and the specimen was resected. Splenic perfusion was confirmed using indocyanine green fluorescence imaging [8]. The operative time was 140 min, with minimal estimated blood loss. The patient was discharged on postoperative day 7 without any complications or recurrence 3 months after surgery. The patient will be followed up without adjuvant therapy using radiological examinations every 3–6 months.

**FIGURE 3:**
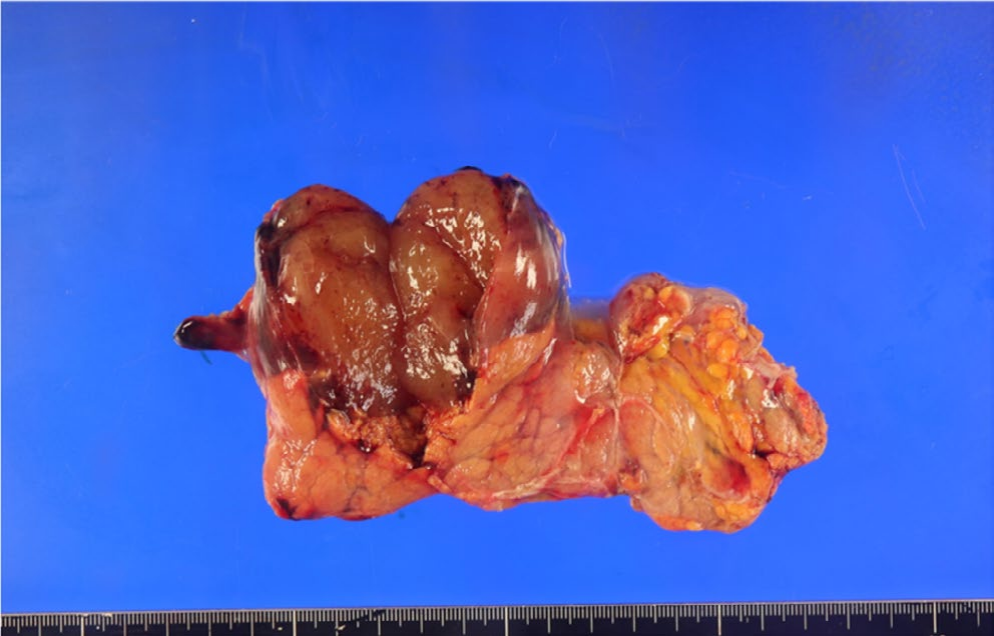
A well‐defined tumorous lesion measuring 60 mm in the pancreatic body. The cut surface shows a myxoid appearance.

**FIGURE 4:**
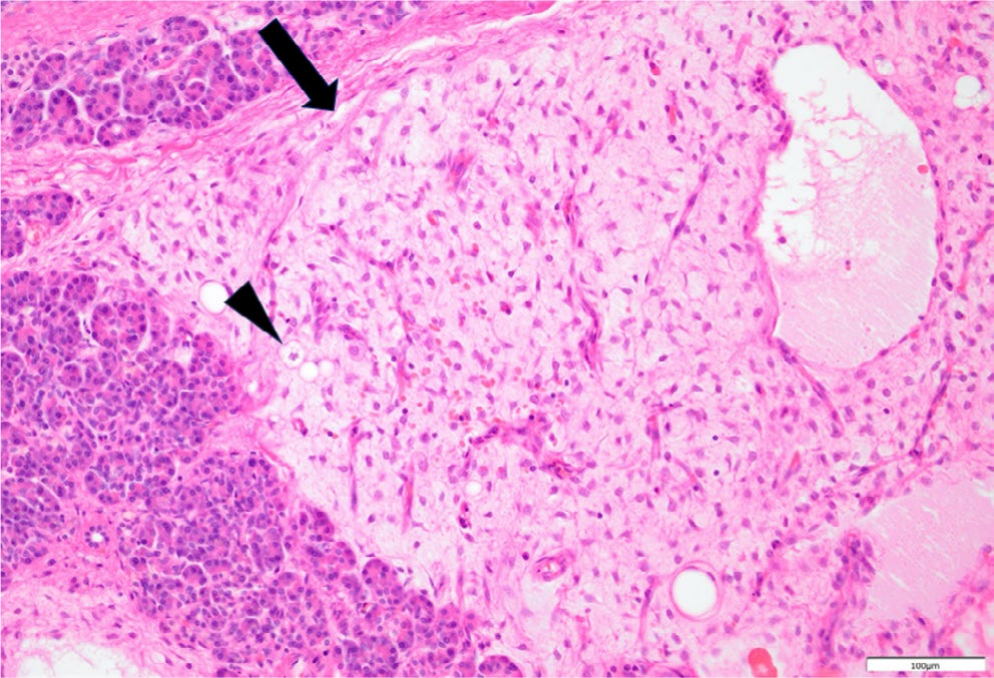
Hematoxylin and eosin staining at 200× magnification. Round‐to‐stellate atypical cells (arrow) proliferating within a myxoid stroma, infiltrating the pancreatic parenchyma and adipose tissue. A few lipoblasts (triangles) can also be seen.

Gross inspection revealed a 60‐mm well‐defined tumor in the pancreatic body. The cut surface of the tumor had a myxoidal appearance (Figure 3). Pathological findings revealed round‐to‐stellate atypical cells proliferating within the myxoid stroma, infiltrating the pancreatic parenchyma and adipose tissue. Most areas showed cystic changes, and a few lipoblasts were observed. No round cell components were observed (Figure 4). Immunohistochemical analysis was positive for DDIT3 and negative for MDM2 and CDK4. Accordingly, the tumor was diagnosed as a pancreatic metastasis of a previously resected MLS in the right thigh. A pathological R0 resection was performed.

## 3 Discussion

The present study demonstrated an unusual recurrence pattern of MLS in the pancreas. To the best of our knowledge, this is the first report of a case of metachronous pancreatic metastasis of MLS that was successfully treated with robotic SPDP. Moreover, minimally invasive surgery is feasible for solitary pancreatic metastases from MLS.

Long‐term outcomes of MLS have been reported to be more favorable than those of other liposarcoma subtypes; however, metastases have been found in approximately one‐third of the patients with MLS [10, 11]. Most STSs tend to metastasize primarily to the lungs and secondarily to other sites. In contrast, MLS has a unique tendency to spread to extrapulmonary sites, including other soft tissue sites and skeletal bones [11]. Moreover, solitary metastasis to an intra‐abdominal organ is rare and pancreatic metastasis of MLS is extremely rare. To date, only two cases of pancreatectomy for pancreatic metastasis of MLS have been reported [5, 6]. Carboni et al. [5] reported a case of isolated pancreatic metastasis 6 years after primary resection treated with pancreaticoduodenectomy. The patient remained disease‐free for 6 months postoperatively [5]. Another case reported by Wang et al. [6] involved solitary pancreatic metastasis 5 years after primary resection, which was managed with central pancreatectomy. After 12 months of disease‐free survival, the patient underwent radical resection for local recurrence of the primary MLS and had a 22 months disease‐free survival [6].

We performed SPDP with resection of the splenic vessels (Warshaw technique) in the present case because wide resection with negative margins is recommended for primary MLS [6]. Considering that the pancreatic tumor was in contact with the splenic vessels, the Warshaw technique was preferred over the Kimura technique. Moreover, retroperitoneal dissection was performed at the level 1 dissection line because there was no tumor invasion into the surrounding tissues [9]. Accordingly, minimally invasive surgery was selected, considering the tumor characteristics and growing evidence of the success of robotic surgery. This case suggested that curative resection should be considered in patients with pancreatic metastases from MLS. Further long‐term follow‐up is required.

In conclusion, this study described an extremely rare case of pancreatic metastasis of MLS in the right thigh that was resected 2 years ago. Successful treatment with robotic SPDP demonstrated that minimally invasive surgery can be indicated for solitary pancreatic metastases of MLS.

## Ethics Statement

Because this was a single report and not a trial or observational research, there was no requirement for ethical approval. Written informed consent was obtained from the patient for publication of this case report and any accompanying images.

## Conflicts of Interest

The authors declare no conflicts of interest.

## Supporting information


**Figure S1.** Magnetic resonance imaging showing primary myxoid liposarcoma in the right thigh, measuring 25 cm in diameter (T2‐weighted sequence).
**Figure S2.** Magnetic resonance imaging showing a 25‐mm tumor in the pancreatic body (T2‐weighted sequence).
**Figure S3.** Magnetic resonance imaging showing an enlarged 60‐mm tumor in the pancreatic body (T2‐weighted sequence).

## Data Availability

The data that support the findings of this study are available on request from the corresponding author. The data are not publicly available due to privacy or ethical restrictions.
