# Supplementary material for: Metachronous Pancreatic Metastasis of Myxoid Liposarcoma Successfully Treated With Robotic Spleen‐Preserving Distal Pancreatectomy With Splenic Vessels Resections: A Case Report
Source: Asian J Endosc Surg. 2025 Apr 22;18(1):e70069. doi: 10.1111/ases.70069 (PMC12014522; doi:10.1111/ases.70069)

**Supplementary material**

**Figure S1** Magnetic resonance imaging showing primary myxoid liposarcoma in the right thigh, measuring 25 cm in diameter (T2-weighted sequence).


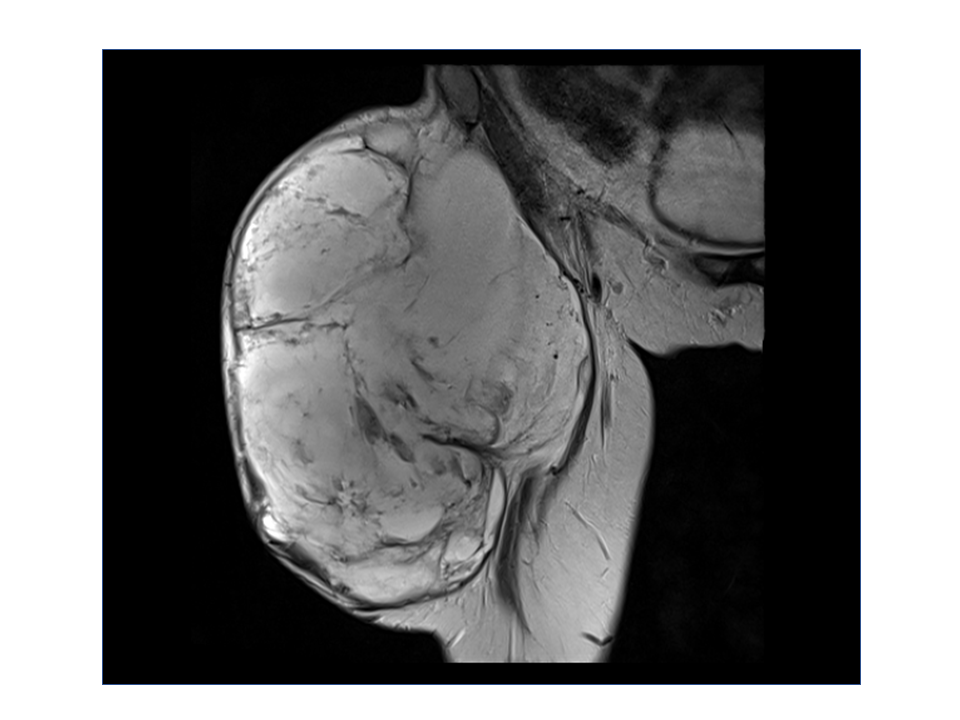


**Figure S2** Magnetic resonance imaging showing a 25-mm tumor in the pancreatic body (T2-weighted sequence).


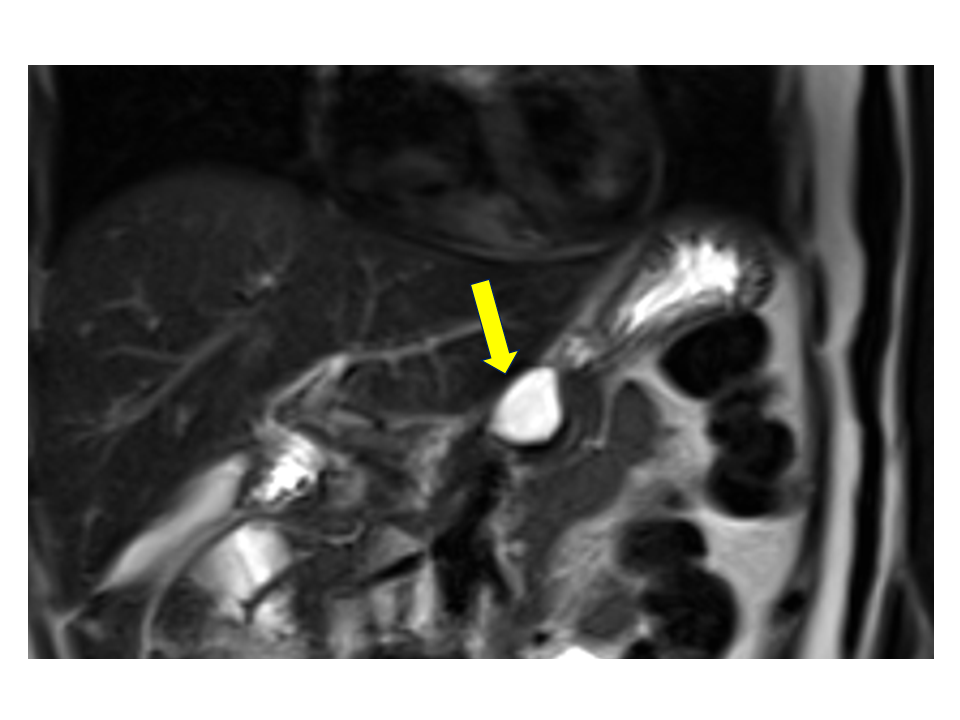


**Figure S3** Magnetic resonance imaging showing an enlarged 60-mm tumor in the pancreatic body (T2-weighted sequence).


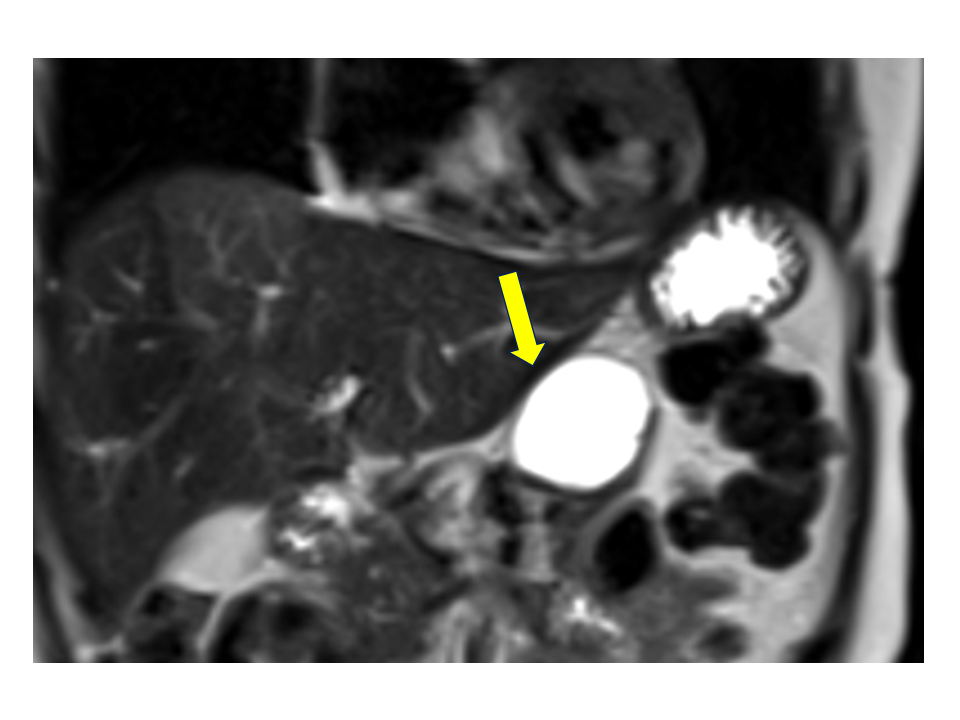

Supplement: Supplementary file 1 — Figure S1. Magnetic resonance imaging showing primary myxoid liposarcoma in the right thigh, measuring 25 cm in diameter (T2‐weighted sequence). Figure S2. Magnetic resonance imaging showing a 25‐mm tumor in the pancreatic body (T2‐weighted sequence). Figure S3. Magnetic resonance imaging showing an enlarged 60‐mm tumor in the pancreatic body (T2‐weighted sequence). [file ASES-18-e70069-s001.docx]
